# Financial Cost Analysis of DREAMS Clinic-Based and Community Programs in Two Provinces of Mozambique, October 2022 to June 2023

**DOI:** 10.3390/tropicalmed11100294

**Published:** 2026-10-16

**Authors:** Mario Songane, Maria Vides, Mahoudo Bonou, Assucênio Chissaque, Amade Suca, Humberto Inguane, Jose Chivite, Meghan Duffy, Sushant Mukherjee

**Affiliations:** 1 Elizabeth Glaser Pediatric AIDS Foundation (EGPAF), Avenida Paulo Samuel Kakhomba, Numero 280, Maputo 1101, Mozambique; 2 Independent Consultant; 3 Elizabeth Glaser Pediatric AIDS Foundation (EGPAF), 1120 19th St. NW, Washington, DC 20036, USA

**Keywords:** DREAMS, costs, adolescents, HIV, Mozambique

## Abstract

The Determined, Resilient, Empowered, AIDS-free, Mentored, and Safe (DREAMS) program delivers community- and clinic-based interventions to reduce HIV vulnerability among adolescent girls and young women (AGYW) aged 15–24 years. We conducted a retrospective programmatic financial cost analysis from the implementing partner perspective using financial and programmatic records from October 2022 to June 2023. We estimated the total costs and cost per AGYW enrolled (cost/AGYW) of DREAMS components in Gaza and Inhambane, Mozambique. A total of 19,477 AGYW were enrolled in Gaza and 3320 in Inhambane from October 2022 to June 2023. Clinic-based components cost US$189,047 in Gaza and US$67,826 in Inhambane, which amount to US$10 and US$20 per AGYW enrolled, respectively. Inhambane’s community component cost US$128,344, and US$39 per AGYW enrolled without Siyakha (an economic package that prepares AGYW to enter the job market). Among Siyakha recipients, the combined community and Siyakha cost was US$466 for 59 AGYW receiving the light package without internship and US$551 for 54 AGYW receiving the full package with internship. Human resources were the largest cost category across components, indicating that staffing requirements are a major determinant of DREAMS implementation costs. Human resources were the main cost driver in both components, highlighting the need to optimize staffing models.

## 1. Introduction

In Mozambique, the prevalence of HIV among youth between 15 and 24 years of age is much higher in females (8%) than males (2.6%) [1]. Adolescent girls and young women (AGYW) have elevated risks of getting infected with HIV due to sex partnerships with much older men, a higher per-act risk of male-to-female transmission than female-to-male, poverty, poor access to the job market and education, and transactional sex [2,3,4,5,6].

The DREAMS (Determined, Resilient, Empowered, AIDS-free, Mentored, and Safe) program was a public–private partnership between the U.S. President’s Emergency Plan for AIDS Relief (PEPFAR) and the Bill & Melinda Gates Foundation, Girl Effect, Gilead Sciences, Johnson & Johnson, and ViiV Healthcare that aimed to reach AGYW aged 10–24 years that were at risk of HIV infection [7,8]. The program started in 2015, and by 2023, 16 countries (15 in sub-Saharan Africa plus Haiti) with a high burden of HIV among AGYW were implementing it [9]. In Mozambique, the DREAMS program started in 2016, and it was coordinated by PEPFAR and implemented through a partnership involving the U.S. Centers for Disease Control and Prevention (CDC), United States Agency for International Development (USAID), implementation staff, the Ministry of Health, and the AGYW themselves and their communities [10].

The DREAMS program delivered a layered package of community- and clinic-based services tailored to the age and needs of adolescent girls and young women (AGYW). For girls aged 10–14 years, community services focused on social asset building and basic financial literacy, while clinic-based activities included educational sessions on sexual and reproductive health and violence mitigation. AGYW aged 15–19 years participated in structured girls’ clubs and basic financial literacy activities and received clinic-based services including condom promotion and provision, health counseling, and HIV testing. Similarly, young women aged 20–24 years participated in structured activities aimed at strengthening life skills and economic awareness and in expanded clinic-based services. This age-differentiated approach combines social, economic, behavioral, and biomedical interventions to address the multiple factors contributing to HIV vulnerability among AGYW (Supplementary Table S1). More specifically, girls aged 10–14 years received 18 social asset-building sessions if out of school and 27 sessions if in school, seven basic financial literacy sessions delivered through the community component, and four educational sessions on sexual and reproductive health and violence mitigation through adolescent- and youth-friendly services (AYFS), which were classified within the clinic-based component. For older adolescents aged 15–19, the program included 12 social asset-building sessions, 9 sessions of financial literacy, and 25 economic strengthening sessions. This age-tailored combination of community empowerment and clinic-based prevention services was designed to progressively address social, behavioral, and biomedical HIV risk factors as AGYW transition through adolescence into young adulthood (Supplementary Table S1) [10].

The community component of DREAMS also included Siyakha, a comprehensive economic strengthening package offered to the most vulnerable AGYW (determined using Vulnerability Assessment Standard Operating Procedures). It was employed to prepare and support them to enter the job market. Key activities of this package included vocational and technical skills training, financial literacy, mentorship, internships, and business start-up support. The Siyakha package started with capacity building and ended with the creation of job opportunities in collaborating companies or with the AGYW starting their own business. Siyakha had two different packages, a light package for AGYW aged 15–17 years (without internship) and a full package for AGYW aged 18 to 24 years (with internship) (see Supplemental Table S2).

DREAMS required major financial commitment. A study in Kenya estimated a cost per AGYW between US$67 and 129 [11]. However, the costs of the program in Mozambique have not been sufficiently analyzed. By quantifying the financial resources required to deliver comprehensive interventions targeting AGYW, cost estimation enables policymakers and implementers to optimize resource allocation and identify funding gaps. Here, we estimated the total costs, from the implementing partner’s perspective, and cost per AGYW enrolled (cost/AGYW) of the clinic-based and community components of DREAMS in the two Mozambican provinces where the Elizabeth Glaser Pediatric AIDS Foundation (EGPAF) implemented DREAMS: Gaza and Inhambane.

## 2. Methods

### 2.1. Study Population

The EGPAF implemented DREAMS in five districts (XaiXai, Chokwe, Limpopo, Chongoene, Guija) in Gaza and one district (Maxixe) in Inhambane. Data were included from AGYW enrolled in DREAMS from October 2022 to June 2023 from 5 health facilities and 3 school corners in Inhambane, as well as from 57 health facilities and 12 school corners in Gaza (Table S1). AGYW were enrolled if they met the DREAMS eligibility criteria, completed the program enrollment process, and were registered as DREAMS beneficiaries in the program records during the study period, irrespective of the number of services or sessions subsequently received or completed. Because DREAMS used a layered service delivery model, an enrolled AGYW could receive both community- and clinic-based services. These components therefore did not represent mutually exclusive beneficiary populations. In Inhambane, the same program enrollment denominator was used for the community and clinic-based component costing rather than summing beneficiaries across the two components. Enrollment counts were retrospectively extracted from routinely maintained DREAMS program records by EGPAF Provincial Advisors in Gaza and Inhambane.

All facilities and school corners were supported by the EGPAF with funding through the PEPFAR.

### 2.2. Costing

We conducted a partial programmatic financial cost analysis from the implementing partner perspective, which estimated financial expenditures incurred by the EGPAF between October 2022 and June 2023 (9 months), during the implementation of DREAMS clinic-based and community components. Resource use was identified retrospectively from EGPAF financial and program records, and financial expenditures were extracted from EGPAF’s financial management system and entered into an Excel-based costing tool. Shared resources were allocated according to the proportion attributable to DREAMS and, where applicable, to the relevant clinic-based or community component. Unit costs were based on the actual amounts paid or recorded in EGPAF’s financial records during the study period. All project costs were converted to U.S. dollars (US$) using the prevailing exchange rate at the time of purchase or payment from Mozambique’s Central Bank (range: US$1 = MZN63.24 to US$1 = MZN63.25).

In Inhambane, we estimated the costs of both clinic-based and community components of DREAMS, whereas in Gaza, we estimated the costs of the clinic-based component (as the community component was supported by another implementer). In Inhambane, both components were funded by the PEPFAR through the CDC and implemented by the EGPAF. Cross-province comparisons were restricted to the clinic-based component only.

#### 2.2.1. Cost Categories

Financial costs were extracted from the EGPAF’s finance management software and input into an Excel cost collection tool in August 2023 by the Finance Officer. Costs were divided in five categories: human resources, travel, supplies, capital (furniture, equipment and training) and, for those eligible, costs for the Siyakha package. We only included programmatic costs from October 2022 to June 2023; we excluded overhead and administrative costs, infrastructure construction or maintenance, clinic operations and care, and costs related to community DREAMS activities in Gaza supported by another implementer.

The personnel for the community component comprised DREAMS Mentors, DREAMS Mentor Supervisors, DREAMS Ambassadors, DREAMS Ambassador Coordinator, DREAMS Economic Facilitators, DREAMS Economic Facilitator Supervisors, DREAMS family dialog facilitators, and Community DREAMS focal points. The personnel for the clinical component comprised Menstrual Hygiene Facilitators, Menstrual Hygiene Facilitator Supervisors, District DREAMS clinical focal points, and Maternal Child Health (MCH) nurses working on specific Adolescent and Youth-Friendly Health Services and school corners. Since these cadres were dedicated to DREAMS full time, their costs were calculated by multiplying the monthly salary by the number of months worked (9 months). The EGPAF did not pay direct costs for clinic operations and medical care provided; these are not included in the cost results.

Allowable costs for the travel (i.e., technical support, supervision) cost category included accommodations, per diem and fuel. Supply costs primarily comprised stationary and printing. Equipment, furniture, and training costs were treated as capital costs.

Equipment and furniture costs were annualized over a five- and ten-year useful life, respectively, with a discount rate of 3% based on the World Health Organization’s (WHO) cost-effectiveness analysis guidelines [12,13]. Costs were annualized by dividing the total cost of equipment or furniture by the annuity as described previously [14,15] and then prorated to the 9-month implementation period by multiplying by 0.75. Training typically occurred once at the beginning when the intervention was rolled out and had a validity of three years; thus, we treated them as capital costs and annualized them over three years, as done previously by others [14,15]. We applied a discount rate of 3% and calculated the annualized costs as described above. Training costs included the venue rent, facilitator fee, training material, allowance/stipend, accommodation, and lunch.

The cost of each component in each province was calculated by adding the total costs of each category. In Inhambane, we also added the cost of Siyakha. For each activity, we calculated the cost per participant by dividing the total cost by the number of participants. The costs were allocated to the light or full package by multiplying the cost per participant by the number of participants. Siyakha was not a universal component delivered to all DREAMS participants. It was an additional economic strengthening intervention designed for a small group of AGYW meeting predefined vulnerability criteria.

#### 2.2.2. Cost per AGYW Enrolled and Sensitivity Analysis

The cost per AGYW enrolled and completed program in each province was calculated by dividing the total 9-month costs of implementing each component of the DREAMS program in each province by the number of AGYW enrolled and that completed the program, respectively, as displayed in the following equations:

(a) Clinic-based component:

$$Cost\;per\;AGYW\;enrolled=\frac{Total\;clinic\;cost}{N}$$

$$Cost\;per\;AGYW\;completed\;program=\frac{Total\;clinic\;cost}{N}$$

(b) Community component (in Inhambane only):

$$Cost\;per\;AGYW\;enrolled=\frac{Total\;community\;cost}{N}+cost\;of\;Siyakha/person$$

Note: The additional cost of Siyakha/person is only applicable for those who are eligible (59 and 54 AGYW who received the light and full package, respectively).

$$Cost\;per\;AGYW\;completed\;program=\frac{Total\;clinic\;cost}{N}$$

where

*N*—total number of AGYW enrolled.

For those eligible, we added the cost per AGYW enrolled for the Siyakha light or full package.

One-way sensitivity analysis was conducted to assess how variations in each cost category affected the cost per AGYW enrolled. Each cost input was varied by applying a ±10% variation range while the others remained the same, as previously done by us and others [16,17]. A ±10% range was selected because no empirical confidence intervals or parameter-specific uncertainty ranges were available for the programmatic and financial inputs. This range provides a pragmatic and symmetric assessment of moderate uncertainty around observed values and is consistent with previous studies on HIV service cost when evaluating uncertainty in resource use and cost parameters [16,17].

#### 2.2.3. Scenario Analysis

Scenario analyses were conducted to assess the robustness of the cost per AGYW enrolled to alternative assumptions regarding program scale, discount rates, and the useful life of capital items. Three sets of scenarios were evaluated against the base case. First, program scale was varied by decreasing or increasing AGYW enrollment by 20%, with human resource costs adjusted proportionally by the same percentage to reflect corresponding changes in staffing requirements, while other cost categories were held constant. Second, alternative discount rates of 1% and 5% were applied, compared with the 3% rate used in the base case. Third, alternative useful lives for capital items were assessed. The lower-useful-life scenario assumed 5 years for furniture, 3 years for equipment, and 2 years for training, compared with 10, 5, and 3 years, respectively, in the base case. The higher-useful-life scenario assumed 15 years for furniture, 7 years for equipment, and 4 years for training. For each scenario, total costs and cost per AGYW enrolled were recalculated while all other assumptions remained unchanged.

## 3. Ethics Statement

This evaluation was implemented under the auspices of the EGPAF’s Patient and Program Outcomes Protocol (PPOP). Permission and ethical clearance to conduct this protocol was obtained from Mozambique’s Ministry of Health institutional review board (IRB) (approval number: CNBS/656/19) and Advarra in the United States. The PPOP is limited to the analysis of secondary data that are routinely collected as part of the standard services and do not pose added risks to the safety or rights of participants. This evaluation did not involve direct interaction with participants, and the ethics review board waived the requirement for informed consent. Only aggregate participant data were used, and no additional participant information was collected outside of the records at the time of data extraction. This project was reviewed in accordance with CDC human research protection procedures and was determined to be non-research.

## 4. Results

### 4.1. Number of AGYW Enrolled and Completed the Program

Between October 2022 and June 2023, a total of 19,477 AGYW were enrolled (13,633, 70%, completed the program) in Gaza and 3320 AGYW were enrolled (1185, 36%, completed the program) in Inhambane.

### 4.2. Siyakha Package

Siyakha is a comprehensive community economic strengthening package that was offered to the most vulnerable AGYW in Inhambane. A total of 59 AGYW were enrolled in the Siyakha light package and 54 in the full package. From October 2022 to June 2023, the total cost of the Siyakha full package was US$27,687 and the cost per AGYW enrolled was US$513, and US$25,206 and US$427 for the light package, respectively (Table 1).

### 4.3. Total Cost and Cost per AGYW Enrolled and Completed DREAMS Program

The total cost of implementing the DREAMS clinic-based component from October 2022 to June 2023 in Inhambane was US$67,826, and US$189,047 in Gaza. The total cost of implementing the DREAMS community component, excluding Siyakha, in Inhambane was US$128,344 (Table 2 and Table S3). The cost per AGYW enrolled in the clinic-based component in Inhambane was US$20, and US$10 in Gaza. The cost per AGYW enrolled in the community component in Inhambane was US$39 per AGYW without Siyakha (a complementary package of the community component), US$551 per AGYW with the Siyakha full package, and US$466 per AGYW with the Siyakha light package (Table 2). The cost per AGYW who completed the program was US$57 and US$14 for the clinic-based components in Inhambane and Gaza, respectively, and US$108 for the community component in Inhambane. In both components and provinces, the largest expenditure was on human resources, which corresponded to over 60% of the total expenditure.

### 4.4. Sensitivity Analysis

Sensitivity analysis was conducted to quantify the effect of parameter uncertainty on cost per AGYW enrolled (Figure 1a,b). A ±10% change in the number of AGYW enrolled resulted in the largest variation in unit costs in both provinces and components. Specifically, in Inhambane, a 10% decrease in enrollment increased the cost per AGYW from US$20 to US$23 in the clinic-based component and from US$39 to US$43 in the community component, while a 10% increase in enrollment reduced costs to US$19 (clinic-based) and US$35 (community). In Gaza (clinic-based component only), a 10% decrease in enrollment increased the cost per AGYW from US$10 to US$11, whereas a 10% increase reduced it to US$9. Across all components, variations in human resource costs had the second-largest impact on unit costs, while changes in supplies, training, travel, and capital costs resulted in relatively smaller fluctuations.

### 4.5. Scenario Analysis

Scenario analysis showed that cost per AGYW was most sensitive to changes in program scale (Table 3). A 20% reduction in enrollment with proportional staff reduction increased cost per AGYW to US$41.04, US$22.34, and US$10.30 for the Inhambane community, Inhambane clinic-based, and Gaza clinic-based components, respectively, while a 20% increase reduced costs to US$37.07, US$19.16, and US$9.31. Changes in discount rates had minimal impact, while shorter useful lives of capital items moderately increased unit costs and longer useful lives reduced them. Overall, the cost estimates were robust to alternative discount rate and capital useful life assumptions.

## 5. Discussion

The current cost analysis of the DREAMS program in Mozambique provides new, detailed evidence on the financial requirements for delivering a layered package of HIV prevention, economic strengthening, and sexual and reproductive health (SRH) services for AGYW. Human resources were the main cost driver in both provinces and components, accounting for more than 60% of total cost. In Inhambane, the cost per AGYW enrolled and completed program in the clinic-based component (US$20 and US$57, respectively) was substantially lower than in the community component (US$39 and US$108, respectively), primarily driven by higher personnel in the community platform. Personnel costs accounted for a larger share of total expenditure in the community component (75%) compared to the clinic-based component (63%), reflecting the labor-intensive nature of community delivery through mentors, facilitators, and supervisors. These differences in cost structure help explain the higher unit cost observed for the community component and highlight the importance of delivery modality in determining program efficiency.

In the community component, 48% (US$46,634) of the personnel costs were spent on DREAMS Mentors (Table S3a) due to the nature, intensity, and delivery modality of its interventions. Community-based DREAMS activities relied on small-group and individualized engagement, including girls’ clubs, safe spaces, family dialog sessions, financial literacy training, and continuous follow-up to ensure retention and completion [2,3,10]. These activities were delivered outside formal health facilities, often across dispersed and rural settings, which increases travel time, supervision needs, and the mentor-to-AGYW ratio required to maintain program quality.

While these findings underscore the inherently labor-intensive nature of community-based DREAMS delivery, they also point to opportunities to optimize human resource models without compromising program quality. Potential strategies include refining mentor-to-AGYW ratios based on activity type and risk profile, introducing differentiated intensity of follow-up (e.g., more intensive support for most vulnerable AGYW or newly enrolled AGYW), and leveraging group-based delivery and task shifting where feasible to improve efficiency [2,18,19].

Furthermore, the costs of the community component varied greatly depending on the inclusion (and type) of the Siyakha package. Although Siyakha provided critical support to the most vulnerable, Siyakha’s cost per AGYW enrolled is much higher and the viability of this program in a time of declining funding for low- and middle-income countries is questionable. Thus, interventions such as Siyakha need to be carefully targeted and evaluated for impact. Nevertheless, the community component may be worth implementing without Siyakha because its package delivers core DREAMS interventions that directly address key social and behavioral drivers of HIV risk among AGYW; however, a cost-effectiveness analysis of DREAMS is required to determine viability. These interventions were particularly important in rural and peri-urban contexts where access to facility-based services is a challenge. In addition, the community component played a critical enabling and complementary role to the clinic-based component, even in the absence of economic strengthening. While Siyakha may provide additional long-term socioeconomic benefits for a subset of highly vulnerable AGYW, its high unit cost warrants careful targeting and separate value-for-money assessment. Nevertheless, the implementation of community-based services of DREAMS has been shown to reduce the incidence of HIV among AGYW, improving health-related and social outcomes [20,21,22].

The clinic-based component was more expensive in total in Gaza (US$189,047) than in Inhambane (US$67,826), but substantially cheaper per AGYW enrolled in Gaza (US$10) than in Inhambane (US$20) because Gaza enrolled far more AGYW (19,477 vs. 3320). The lower unit cost in Gaza may reflect economies of scale, but this interpretation is limited by differences in implementation structure and included cost categories. In both provinces, human resources dominated clinic-based costs (about 76% in Gaza and 63% in Inhambane), suggesting that staffing optimization can lower the cost per AGYW.

In addition to serving AGYW enrolled in DREAMS, DREAMS-supported adolescent- and youth-friendly clinical platforms could also benefit adolescents accessing services through schools and health facilities who were not enrolled as DREAMS beneficiaries [23]. The reach of the DREAMS program extended beyond the target DREAMS beneficiaries listed [24] and an analysis of the impact of DREAMS in both youths enrolled and not enrolled in the prevention of HIV acquisition, violence mitigation (VM), and improving social and economic outcomes would provide important information on the actual impact of the DREAMS program in the community as a whole. A quasi-experimental or matched comparison design could provide evidence on outcomes such as early marriage, school retention, employment, and HIV incidence, enabling a cost-effectiveness assessment of DREAMS and its subcomponents, including Siyakha.

Scenario analysis showed that estimates were relatively robust to alternative discount rate and capital useful life assumptions, while changes in program scale (personnel and enrollment) had a greater effect on unit costs. As shown in the sensitivity analysis, changes in the number of AGYW enrolled had a substantial effect on the estimated cost per AGYW, with a ±10% variation in enrollment resulting in corresponding changes in unit costs across both components and provinces. However, these findings have to be interpreted with caution because in practice, expanding enrollment would likely require additional investments in personnel, supervision, and outreach, particularly for community-based delivery models that rely on maintaining appropriate mentor-to-AGYW ratios to ensure quality of services. Furthermore, the costs of enrollment activities (i.e., community mobilization, stakeholder engagement, and awareness campaigns) that were not included in this analysis would also need to be considered when assessing the cost implications of program expansion.

## 6. Conclusions

In this study, the total costs and cost per AGYW enrolled across clinic-based and community components, including Siyakha, were estimated, and partial programmatic cost estimates are provided, which may support preliminary budgeting and help identify major cost drivers. Human resources were the main cost driver in both components, highlighting the need to optimize staffing models and mentor ratios. Average costs per AGYW decline with higher enrolment, highlighting the efficiency gains possible through scale-up. Investment in DREAMS may improve the employability of AGYW; however, the inclusion of Siyakha packages requires further cost–benefit analysis.

## 7. Limitations

This study had some limitations that should be considered when interpreting the findings. First, the analysis was conducted in only two provinces (Gaza and Inhambane) and for a limited implementation period of nine months, which may limit the generalizability of the cost estimates to other scenarios. Second, costs were estimated from a programmatic perspective and excluded some relevant inputs such as overhead and administrative costs, health facility operating costs, care costs, infrastructure construction and maintenance, and the costs of key enrollment-related activities such as community mobilization, stakeholder engagement, and demand creation. Consequently, the reported costs are likely to underestimate the resources required to replicate the complete DREAMS program. Third, we do not have reliable data with which to calculate the cost per AGYW receiving the full layered intervention or cost per service/session delivered. Four, detailed service-level output data were not available for this retrospective analysis. We were therefore unable to quantify the actual number of sessions or modules completed, clinic visits, HIV tests, PrEP referrals, contraceptive services, or other individual services delivered during the study period. The estimates should therefore not be interpreted as measures of service delivery intensity or technical efficiency. Future economic evaluations of DREAMS should link expenditure data with service-level utilization and completion data to estimate costs per service delivered and per completed package and to assess differences in technical efficiency across delivery models.

## Figures and Tables

**Figure 1:**
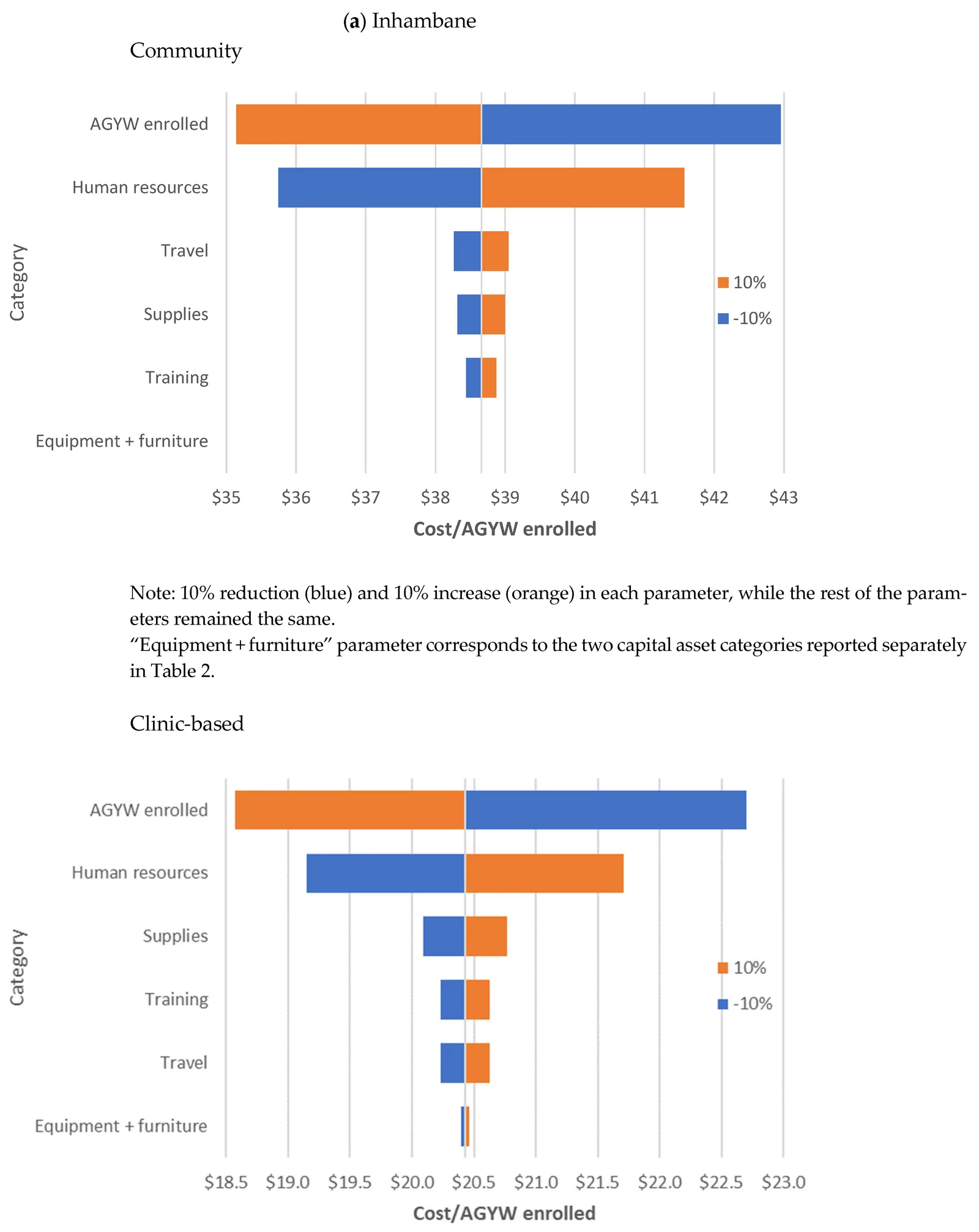

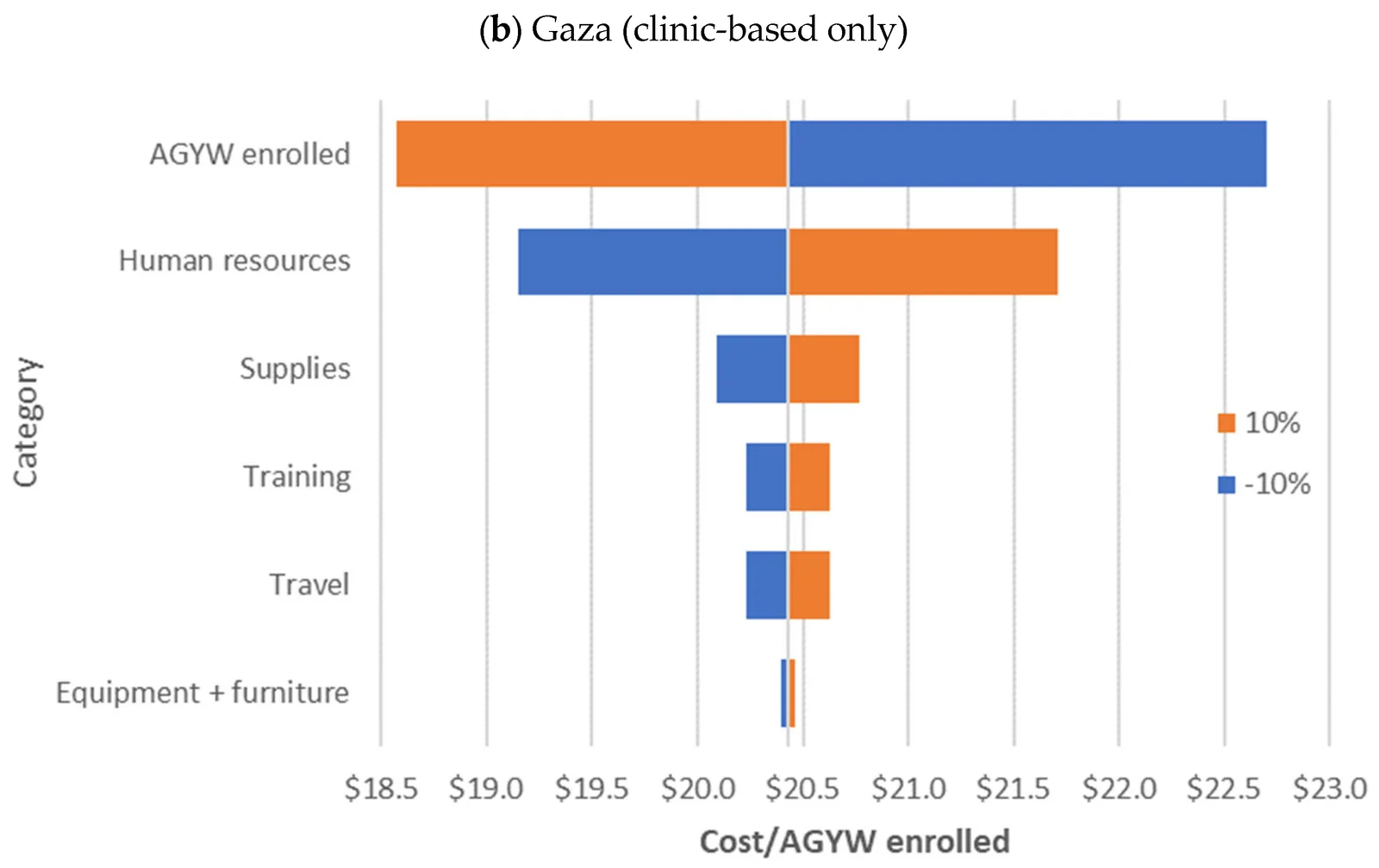
Tornado plot of one-way sensitivity analysis: cost per AGYW enrolled in (**a**) Inhambane and (**b**) Gaza.

**Table 1:** Total costs and cost per AGYW of Siyakha packages.

Activity	Item	Light (*n* = 59 AGYW) (US$)		Full (*n* = 54 AGYW) (US$)	
		Cost/AGYW	Total	Cost/AGYW	Total
Activity 1. Siyakha Model Training of Trainers for IP Staff	Curriculum adaptation	4	209	4	191
	Transport reimbursements	3	193	3	176
	Venue hire	8	466	8	427
	Lunch, stationary, per diems and accommodation	4	208	4	190
	Labor market assessment: LMA Feedback and Inception meetings with stakeholders	2	94	2	86
	Training consumables	1	31	1	29
	Printing	1	79	1	72
	Per diem for local government personnel	0.4	21	0.4	19
	Subtotal	22	1301	22	1191
Activity 2: Selection of Young Women Participants	Enrolment tools	0.1	8	0.1	7
	Social contract forms	0.1	4	0.1	4
	Subtotal	0.2	12	0.2	11
Activity 3. Cascade Foundational Skills Training for AGYW	Training materials	4	236	4	216
	Student manual printing	10	590	10	540
	Subtotal	14	826	14	756
Activity 4: Vocational Training for AGYW	Vocational training fees	78	4602	78	4212
	Vocational training tools and protective clothing	30	1770	30	1620
	Stipends for transport	47	2773	47	2538
	Graduation certificate printing	1	30	1	27
	Subtotal	156	9175	156	8397
Activity 5: Social Asset Building Training	Training materials	4	236	4	216
	Venue hire (when needed)	8	495	8	453
	Conference fees	7	385	7	353
	Subtotal	19	1116	19	1022
Activity 6: Exit Review Meetings	Venue hire	6	371	6	340
	Lunch and stationary	10	605	10	553
	Subtotal	17	976	17	893
Activity 7: Internships	Internship MoU printing			1	27
	Transport reimbursements			70	3780
	Internship tools			15	810
	Subtotal			86	4617
Activity 8: Entrepreneurship Support	Start-up kits for businesses	200	11,800	200	10,800
**Total**		**427**	**25,206**	**513**	**27,687**

**Table 2:** Total cost of DREAMS and cost per AGYW enrolled in (**a**) Inhambane (community and clinic-based components) and (**b**) Gaza (clinic-based), in US$ and percentage of the total.

(**a**) Inhambane					
**Category**	**Sub-Category**	**Community**		**Clinic-Based**	
		**Cost (US$)**	**%**	**Cost (US$)**	**%**
Human resources		96,719	75.4	42,515	62.7
Travel		13,013	10.1	6531	9.6
Supplies	Stationary	8562	6.7	6667	9.8
	Printing	2733	2.1	4492	6.6
	Subtotal	11,294	8.8	11,159	16.5
Cost prorated to 9-month	Furniture	116	0.1	855	1.3
	Equipment		0.0	210	0.3
	Training	7202	5.6	6556	9.7
	Subtotal	7318	5.7	7622	11.2
Total 9-month cost		**128,344**	**100.0**	**67,826**	**100.0**
AGYW enrolled		**3320**		**3320**	
AGYW-completed program		**1185**		**1185**	
Cost per AGYW enrolled	Base community package	39		20	
	Incremental Siyakha light	427			
	Combined community + Siyakha light	466			
	Incremental Siyakha full	513			
	Combined community + Siyakha full	451			
**Cost per AGYW–completed program**		108		57.24	
(**b**) Inhambane					
**Category**	**Sub-category**	**Cost (US$)**		**%**	
Human resources		142,994		75.6	
Travel		18,138		9.6	
**Supplies**	Stationary	4262		2.3	
	Printing	10,346		5.5	
	Subtotal	14,608		7.6	
**Capital costs**	Furniture	914		0.5	
	Equipment	254		0.1	
	Training	12,140		6.4	
	Subtotal	13,308		7.0	
Total 9-monthcost		**189,047**		**100.0**	
# AGYW enrolled		19,477			
# AGYW–completed program		13,634			
Cost per AGYW enrolled		10			
**Cost per AGYW–completed program**		14			

**Table 3:** Scenario analysis of the effect of alternative enrollment and staffing levels, discount rates, and capital useful life assumptions on cost per AGYW enrolled.

Parameter	Scenario	Inhambane Community (US$)	Inhambane Clinic-Based (US$)	Gaza Clinic-Based (US$)
Human resources and enrollment	Base case	38.66	20.43	9.71
	−20%	41.04	22.34	10.30
	20%	37.07	19.16	9.31
Discount rate	Base case (3%)	38.66	20.43	9.71
	1%	38.57	20.33	9.68
	5%	38.75	20.54	9.74
Useful life of capital	Base case	38.66	20.43	9.71
	Lower *	39.73	21.64	10.05
	Higher **	38.13	19.87	9.54

* Furniture (5 years), equipment (3 years), training (2 years); ** furniture (15 years), equipment (7 years), training (4 years).

## Data Availability

The data analyzed in this study were derived from routinely collected DREAMS programmatic and financial records from the Elizabeth Glaser Pediatric AIDS Foundation (EGPAF) in Mozambique. The datasets are not publicly available because they contain programmatic and financial information subject to organizational and donor data-sharing requirements. De-identified data supporting the findings of this study may be made available by the corresponding author upon reasonable request and subject to approval by the EGPAF and applicable data-sharing requirements.

## Supplementary Materials

The following supporting information can be downloaded at: https://www.mdpi.com/article/10.3390/tropicalmed11100294/s1, Table S1. Community and clinic-based services provided disaggregated by age. Table S2. Components of the Siyakha packages. Table S3. Breakdown of cost per category, province and Determined, Resilient, Empowered, AIDS-free, Mentored, and Safe (DREAMS) component: (a) personnel, (b) travel, (c) training, (d) equipment and furniture.
