# Supplementary material for: Financial Cost Analysis of DREAMS Clinic-Based and Community Programs in Two Provinces of Mozambique, October 2022 to June 2023
Source: Trop Med Infect Dis. 2026 Oct 16;11(10):294. doi: 10.3390/tropicalmed11100294 (PMC13709781; doi:10.3390/tropicalmed11100294)
Supplement: Supplementary file 1 [file tropicalmed-11-00294-s001.zip › tropicalmed-4390770-supplementary.pdf]

SUPPLEMENTARY INFORMATION

Table S1. Community and clinic-based services provided disaggregated by age [1].

10 to 14 Years	15 to 19 Years	20 to 24 Years
Community Services	Community Services	Community Services
1. Social resources		
2. Basic financial literacy	1. Guide to facilitate girls clubs	1. Guide to facilitate girls clubs
Clinic-based services	2. Basic financial literacy	2. Basic Financial Literacy
1. Educational sessions covering sexual, reproductive health, violence mitigation (VM)	Clinic-based services	Clinic-based services
	1. Promotion and/or Provision of Condoms and contraceptives<br>2. health counseling and testing<br>Siyakha light package	1. Promotion and Provision of Condoms<br>2. health counseling and testing<br>Siyakha full package

Table S2. Components of the Siyakha packages.

Activity	Package	
	Light	Full
Target group	15-17 years old	>18 years
Duration	3-5 months	6-9 months
Intro training (financial literacy, leadership, preparation for job market)	Included	Included
2 months vocational training	Included	Included
Capacity building on social assets	Included	Included
3 months internship	Not included	Included
Start-up kits for businesses	Included	Included
Follow up after graduation	Included	Included

Table S3. Breakdown of cost per category, province and Determined, Resilient, Empowered, AIDS-free, Mentored, and Safe (DREAMS) component: (a) personnel, (b) travel, (c) training, (d) equipment and furniture.

a) Personnel

Clinical component			
**Province**	**Cadre**	**Number of personnel**	**Cost (US$)**
Gaza	Absorb menstrual hygiene package facilitators (DREAMS)	25	43,990
Gaza	Absorb menstrual hygiene package facilitators Supervisor (DREAMS)	3	6,385
Gaza	District DREAMS clinical focal point (Maternal Child Health, MCH, nurse)	5	82,512
Gaza	MCH nurse working on specific AYFS and school corners	3	10,107
	**Total**	**36**	**142,994**
Inhambane	Absorb menstrual hygiene package facilitators (Dreams)	4	7,038

Inhambane	Absorb menstrual hygiene package facilitators Supervisor (Dreams)	1	2,128
Inhambane	District DREAMS clinical focal point (MCH nurse)	1	16,502
Inhambane	MCH nurse working on specific AYFS and school corners	5	16,846
	**Total**	**11**	**42,515**

	**Community component**		
Inhambane	Dreams Mentor	40	46,634
Inhambane	Dreams Mentor Supervisor	4	8,528
Inhambane	DREAMS Ambassador	2	2,332
Inhambane	DREAMS Ambassador Coordinator	1	2,137
Inhambane	Dreams Economic Facilitator	12	13,990
Inhambane	Dreams Economic Facilitator Supervisor	2	4,264
Inhambane	Dreams Family dialog facilitator	2	2,332
Inhambane	Community DREAMS Focal Point	1	16,502
	**Total**	**64**	**96,719**

b) Travel

	**Clinical component**	
**Province**	**Motive**	**Cost (US$)**
**Gaza**	DREAMS provincial visits and Technical Support	1,944
	DREAMS Joint supervision	972
	Adolescent and youth-friendly services (AYFS) protocol quality assessment training	972
	Semester DREAMS Joint partner meeting	4850
	National Supervision	5,640
	Incentives for Mobile brigades for formal and informal working place	3,760
	**Total**	**18,138**
**Inhambane**	DREAMS provincial visits and technical support	1,356
	DREAMS Joint supervision	548
	AYFS protocol quality assessment training	548
	Semester DREAMS Joint partner meeting	2,425
	Quarterly joint supervisory visits with to sites	1,354
	Incentives for Mobile brigades for formal and informal working place	300
	**Total**	**6,531**

	**Community component**	
**Inhambane**	DREAMS provincial visits and Technical Support	1,356
	DREAMS Joint supervision	548

	AYFS protocol quality assessment training	548
	Go girl/go student*	808
	Girl Roster (training of trainers)**	548
	Financial literacy	808
	Supervision	808
	Community engagement	808
	Semester DREAMS Joint partner meeting	2,425
	Semester Siyakha Joint partner meeting	2,194
	Workshop to revise DREAMS national guidelines	808
	Quarterly joint supervisory visits with partners for DREAMS	1,354
	**Total**	**13,013**

* Go girl/go student sstructured social asset–building interventions for AGYW in-school and out-of-school.
**Girl Roster is a database used to manage enrolment, service delivery, and monitoring of DREAMS interventions.

c) Training

Clinical component		
**Province**	**Training**	**Cost (US$)**
**Gaza**	Pre-exposure prophylaxis (PrEP) package for DREAMS MCH and DREAMS Focal Point	555
	VM- Basic package and integrated care package for post-violence care for MCH nurse and DREAMS Focal Point	1,880
	Layering In-Job training DREAMS team	1,556
	DREAMS district training	8,584
	Mentor teacher training	9,023
	School activists training (adolescents to support demand creation)	14,433
	DREAMS quality assessment training	450
	Training adolescent and youth-friendly services (AYFS) package for clinical providers in DREAMS districts	9,304
	**Total**	**45,785**
**Inhambane**	PrEP package for DREAMS MCH and DREAMS Focal Point	1,211
	VM- Basic package integrated care package for post-violence care for DREAMS MCH and and DREAMS Focal Point	1,668
	Layering In-Job training DREAMS team (DLT 2.0)	2,142
	DREAMS district training	275
	Mentor teacher training	5,745
	School activists training (adolescents to support demand creation)	1,298
	DREAMS quality assessment training	581
	Training on AYFS package for clinical providers in DREAMS districts	11,808
	**Total**	24,727

Community component		
**Inhambane**	PrEP package for DREAMS mentors	2,822

Layering In-Job training DREAMS team (DLT 2.0)	2,142
DREAMS district training	275
Mentor teacher training	5,745
School activists training	1,298
DREAMS quality assessment training	581
Go girl/Go student training of trainers	44
Go girl/Go student training	5,046
Financial literacy training of trainers	88
Financial literacy training	4,316
Training on community engagement	4,887
**Total**	27,163

d) Equipment and furniture

		Total cost (US$)	
**Component**	**Furniture**	**Gaza**	**Inhambane**
Clinical	Metallic Cabinet	1,379	2,757
Clinical	Cabinet for drugs	1,379	1,034
Clinical	Steady Office Chairs	376	376
Clinical	Examination Table	846	846
Clinical	Round Table For Adults	329	1,316
Clinical	Chairs For Adults Table	799	533
Clinical	Gynaecological Marquise	1,880	1,253
Clinical	Gynaecological marquise chair	587	392
Clinical	Anatomical models of penis	1,128	376
Clinical	Anatomical models of vagina	1,692	846
	Subtotal clinical	10,395	9,729
Community	White Board	0	1,316
	**Total**	10,395	11,045

		Total cost (US$)	
**Component**	**Equipment**	**Gaza**	**Inhambane**
Clinical	Adult Balance With Altimeter	1,081	721
Clinical	Electronic sphygmomanometer	470	188
Clinical	TV 32'	0	376
	**Total**	**1,551**	**1,285**

References

1. PEPFAR. Preventing HIV in Adolescent Girls and Young Women - Guidance for PEPFAR Country Teams on the DREAMS Partnership [Internet]. 2015. Available from: https://www.unicef.org/childrenandaids/media/2961/file/Preventing HIV in adolescent-girls-

and-young-women-guidance-PEPFAR country-teams-DREAMS-partnership.pdf
